# Supplementary figures and images for: Bridging the gap: organotypic models to study late-onset group B streptococcus infection
Source: Microbiol Spectr. 2026 Apr 22;14(6):e02316-25. doi: 10.1128/spectrum.02316-25 (PMC13228084; doi:10.1128/spectrum.02316-25)

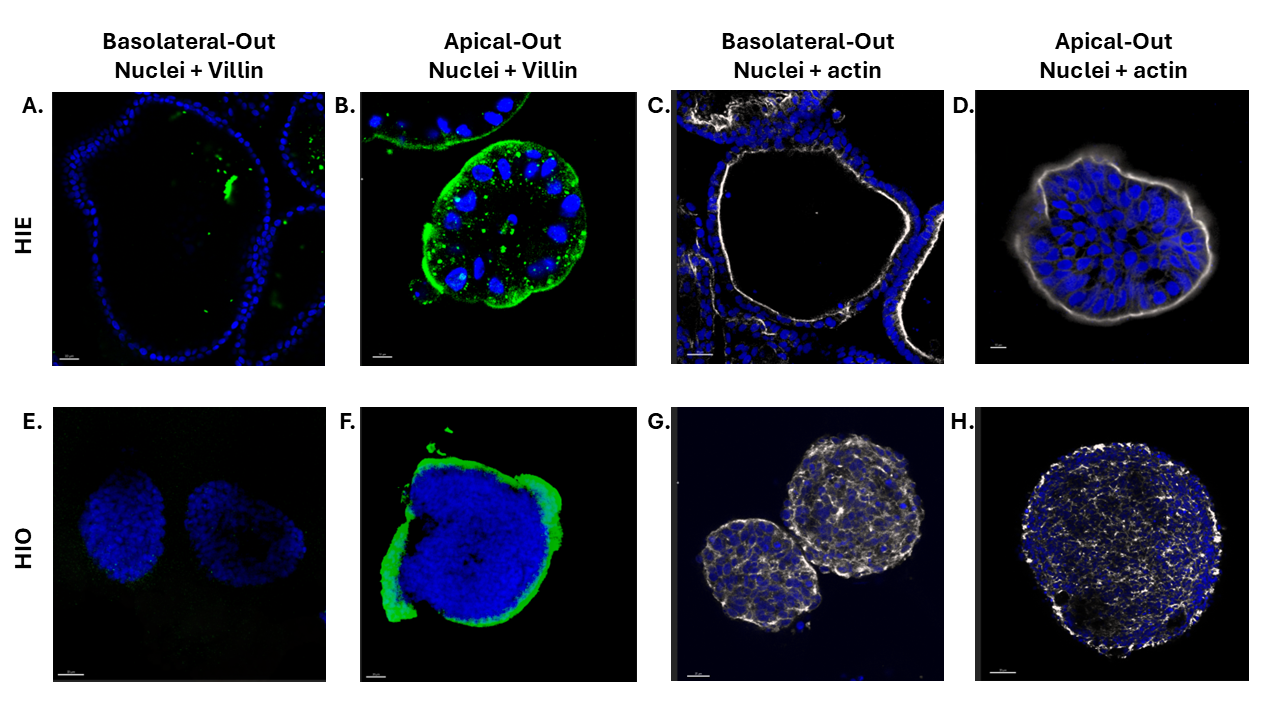

Supplement: Fig. S1 — Polarity reversal controls. [file spectrum.02316-25-s0001.tif]

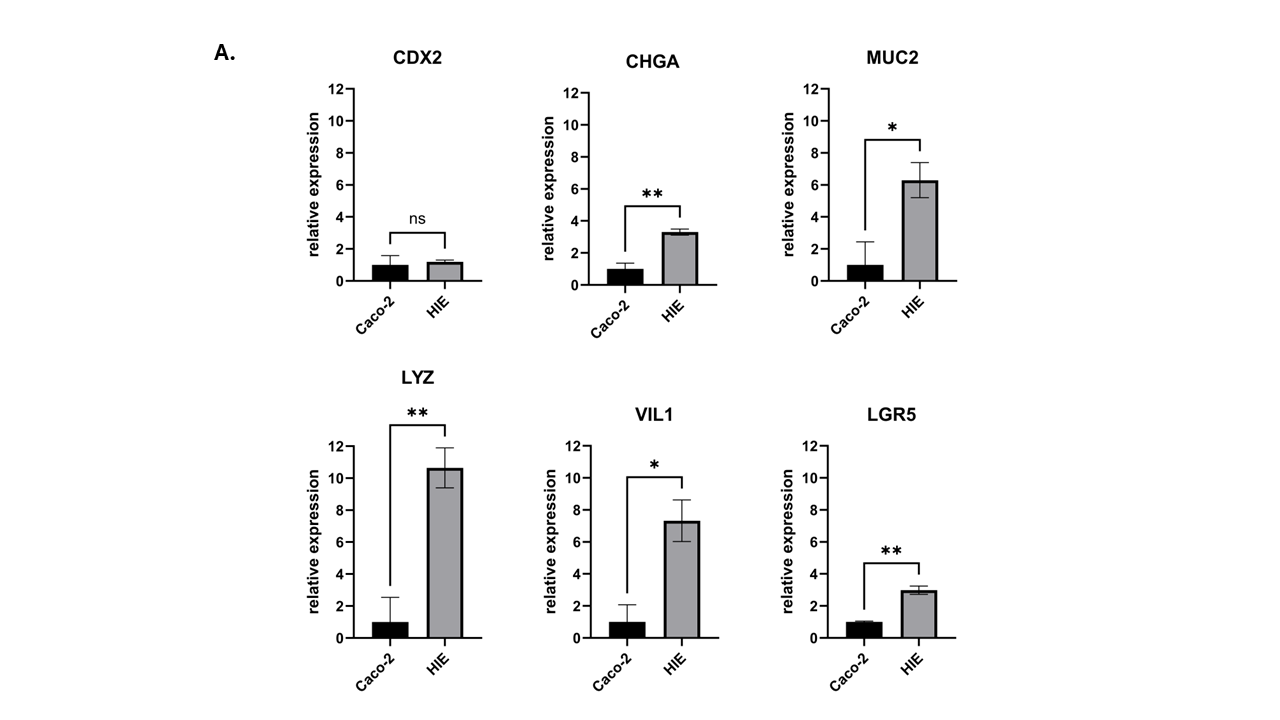

Supplement: Fig. S2 — HIE cell type validation. [file spectrum.02316-25-s0002.tif]

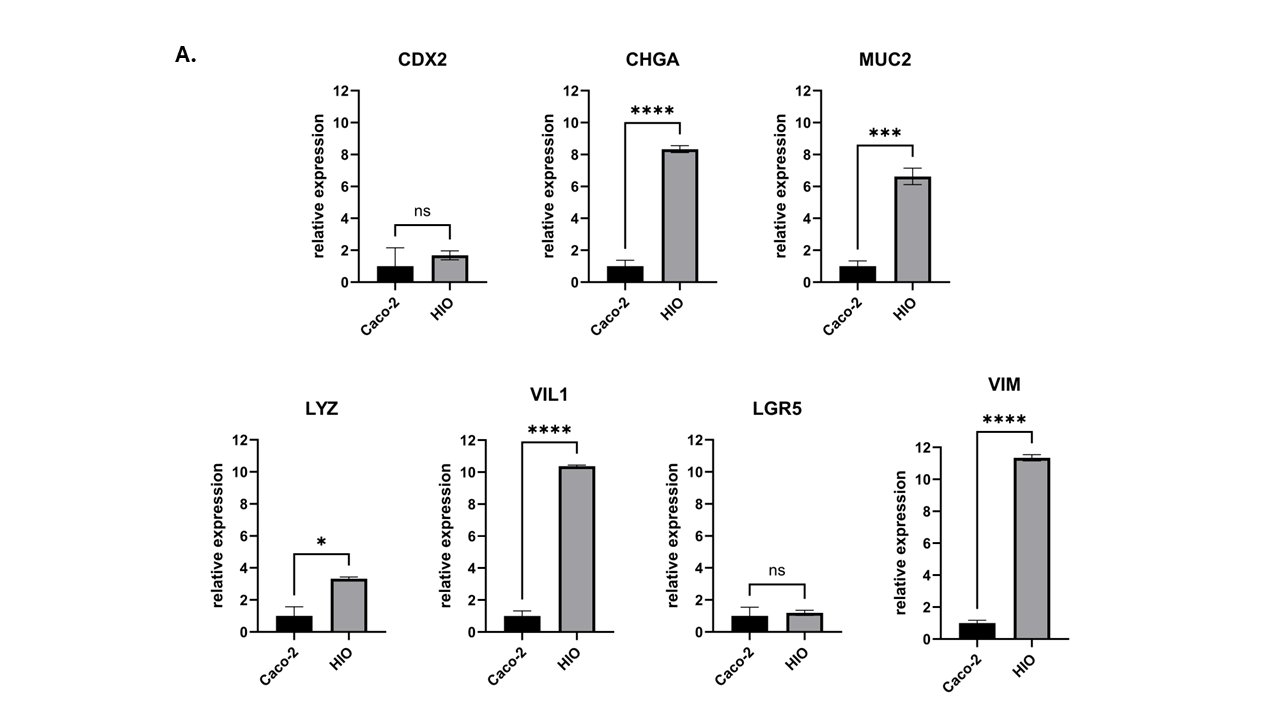

Supplement: Fig. S3 — HIO cell type validation. [file spectrum.02316-25-s0003.tif]
