# Supplementary material for: Bridging the gap: organotypic models to study late-onset group B streptococcus infection
Source: Microbiol Spectr. 2026 Apr 22;14(6):e02316-25. doi: 10.1128/spectrum.02316-25 (PMC13228084; doi:10.1128/spectrum.02316-25)
Supplement: Supplemental material — Supplemental figure legends. [file spectrum.02316-25-s0004.docx]

**Figure S1. Controls for polarity reversal confirmation.** HIE (top row) and HIO (bottom row) basolateral-out and apical-out staining of nuclei [Blue = DAPI], villin [green = anti-mouse IgG Alexa Fluor 488] and F-actin [White = Phalloidin Alexa Fluor™ 488] to visualize polarity orientation. Scale bars HIE top row (left to right): 30 µm, 10 µm, 30 µm, 10 µm. Scale bars HIO bottom row (left to right): 30 µm, 30 µm, 20 µm, 50 µm.

**Figure S2. Validation of presence of cell types within immature intestinal tissue derived human intestinal enteroids.** (**A**) relative gene expression of cell type markers for Caco-2 cells and HIE normalized to the housekeeping gene *GAPDH: CDX2* (enterocytes), *CHGA* (enteroendocrine cells), *MUC2* (goblet cells), *LYZ* (Paneth cells), *VIL1* (villin-apical border), and *LGR5* (intestinal stem cells). Caco-2 cells were used as an undifferentiated control. [unpaired t test, **p<0.009, *p<0.04, ns=nonsignificant]

**Figure S3. Validation of presence of cell types within iPSC derived human intestinal organoids.** (**A**) relative gene expression of cell type markers for Caco-2 cells and HIO normalized to the housekeeping gene *GAPDH: CDX2* (enterocytes), *CHGA* (enteroendocrine cells), *MUC2* (goblet cells), *LYZ* (Paneth cells), *VIL1* (villin-apical border), *LGR5* (intestinal stem cells) and *VIM* (mesenchymal cells). Caco-2 cells were used as an undifferentiated control. [unpaired t test, ****p<0.0001, ***p<0.0008, *p<0.02, ns=nonsignificant]
